# NOTCH1 Gain of Function in Germ Cells Causes Failure of Spermatogenesis in Male Mice

**DOI:** 10.1371/journal.pone.0071213

**Published:** 2013-07-30

**Authors:** Zaohua Huang, Bryan Rivas, Alexander I. Agoulnik

**Affiliations:** 1 Department of Human and Molecular Genetics, Herbert Wertheim College of Medicine, Florida International University, Miami, Florida, United States of America; 2 Department of Obstetrics and Gynecology, Baylor College of Medicine, Houston, Texas, United States of America; Clermont Université, France

## Abstract

NOTCH1 is a member of the NOTCH receptor family, a group of single-pass trans-membrane receptors. NOTCH signaling is highly conserved in evolution and mediates communication between adjacent cells. NOTCH receptors have been implicated in cell fate determination, as well as maintenance and differentiation of stem cells. In the mammalian testis expression of NOTCH1 in somatic and germ cells has been demonstrated, however its role in spermatogenesis was not clear. To study the significance of NOTCH1 in germ cells, we applied a cre/loxP approach in mice to induce NOTCH1 gain- or loss-of function specifically in male germ cells. Using a *Stra8-icre* transgene we produced mice with conditional activation of the NOTCH1 intracellular domain (NICD) in germ cells. Spermatogenesis in these mutants was progressively affected with age, resulting in decreased testis weight and sperm count. Analysis of downstream target genes of NOTCH1 signaling showed an increased expression of *Hes5*, with a reduction of the spermatogonial differentiation marker, *Neurog3* expression in the mutant testis. Apoptosis was significantly increased in mouse germ cells with the corresponding elevation of pro-apoptotic *Trp53* and *Trp63* genes' expression. We also showed that the conditional germ cell-specific ablation of *Notch1* had no effect on spermatogenesis or male fertility. Our data suggest the importance of NOTCH signaling regulation in male germ cells for their survival and differentiation.

## Introduction

NOTCH is a group of trans-membrane receptors which play important roles in various developmental processes and diseases. NOTCH signaling mediates juxtacrine cellular communications and is essential for cell fate determination, embryonic patterning, and development. In mice, a disruption of NOTCH signaling results in abnormal cell differentiation and early embryonic lethality [1], [2]. There are four mammalian NOTCH (NOTCH1-4) receptors, each exhibiting a distinct pattern of expression. All NOTCH receptors have a big extracellular ligand binding domain, a juxtamembrane domain for hetero-dimerization, and an intra- cellular domain for signal transduction. Formation of NOTCH receptor/ligand complex triggers cleavage of the juxtamembrane region of NOTCH receptor by γ-secretase and release of its activated form, NOTCH intracellular domain (NICD). NICD translocates to the nucleus and forms a transcriptionally active complex with the DNA-binding transcription factor CSL (CBF1, Suppressor of Hairless, Lag-1) and the coactivator Mastermind (Mam). NICD converts CSL from a transcription repressor to a transcriptional activator and thus induces the expression of multiple downstream genes [3]. The best characterized among them are the members of the basic helix-loop-helix (bHLH), hairy/enhancer of split (HES) family and the cell cycle regulator p21 (TRP21).

Spermatogenesis is a highly coordinated cell differentiation process in mammals. During the early postnatal period, spermatogonial stem cells (SSCs) originate from pro-spermatogonial gonocyte cells. In rodents, this occurs within the first 6 days, and in primates, within several months after birth [4]. As recently shown, the overexpression of NICD in mouse embryonic Sertoli cells causes the gonocytes to exit from their undifferentiated and quiescent state prematurely [5]. SSCs are located on the basal membrane of testicular seminiferous tubules and either undergo self-renewal or give rise to differentiating spermatogonia. The cell-fate decision is regulated by a milieu of growth factors supplied primarily by Sertoli as well as other somatic cells within SSC niche in the testis [4]. In mice, A_s_ (A-single) spermatogonia populations are believed to represent SSCs. Upon entering differentiation, A_s_ cells produce progenitor A_pr_ (paired) and subtypes of A_al_ (aligned) spermatogonia. Recent studies have reported that the progenitor spermatogonia may revert spontaneously to pluripotent SSCs [6]. However, under normal circumstances, the majority of these cells differentiate into A1, A2, A3, A4, intermediate, and, finally, B spermatogonia. PLZF, OCT4, NANOS2, NEUROG3, GFRα1, RET, FGFR2, ID4 and other markers were found to be highly expressed in SSC populations and were identified as the factors intrinsically required for the maintenance or differentiation of SSCs.

It is established that in Caenorhabditis elegans, Drosophila, and Xenopus, NOTCH signaling is crucial for germ cell development, especially for the formation of the germ stem cell niche and germ stem cell pool maintenance and migration [7]–[10]. In the mammalian testis the expression of different NOTCH family members and their ligands have been described in both testicular somatic cells as well as in male germ cells, specifically in spermatogonia cells [11]–[13]. Some reports suggested that NOTCH1 signaling might promote murine SSC differentiation. Indeed, treatment of SSCs with the SSC maintenance factor GDNF resulted in an increased expression of NUMB, contributing to NICD degradation and the consequent inactivation of NOTCH target genes [14]. Recently, the inactivation of NOTCH signaling in mouse germ cells was achieved using a *Stra8-icre* transgene through the conditional deletion of *Notch1* or deletion of *Pofut1*, a member of canonical NOTCH signaling [15]. Loss of these two genes in germ cells had no effect on testicular weight, histology, or male fertility. However, due to the redundancy of NOTCH receptors' expression or other factors that may compensate for the absence of POFUT1 the loss-of function experiments might be misleading. An alternative approach – the analysis of constitutive NOTCH signaling in spermatogenic cells, has not been analyzed to date.

In the present study, we applied the cre/loxP approach to produce conditional gain- or loss-of-function of NOTCH1 signaling in postnatal mouse male germ cells. In both cases we used the same *Stra8-icre* transgene, which is expressed in early spermatogonial cells beginning from postnatal day 3. The *Notch1* knockout in germ cells had no effect on spermatogenesis. The activation of NOTCH signaling caused a reduction in sperm counts and a progressive loss of testis weight with age. There was a significant increase of germ cell apoptosis in the mutant testis. The NICD gain-of-function was associated with an increased expression of the NOTCH target gene *Hes5* and a decrease of *Neurog3* (Neurogenin 3) expression. We suggest that regulation of NOTCH signaling in germ cells is important for early spermatogonial cell differentiation in the mutant testis.

## Materials and Methods

### Ethics Statement

This study was carried out in strict accordance with the recommendations in the Guide for the Care and Use of Laboratory Animals of the National Institutes of Health. The protocol was approved by the Institutional Animal Care and Use Committee of Florida International University (IACUC Protocol Numbers: 09–003 and 12–006).

### Production of mice with germ cell specific activation and deletion of *Notch1*

All mutant mouse lines were purchased from The Jackson Laboratory, Bar Harbor, ME. The *Gt(ROSA)26Sor^tm1(Notch1)Dam/^*J mice contain a DNA fragment encoding an intracellular portion of the mouse *Notch1* gene (amino acids 1749–2293), but lack the c-terminal PEST domain, inserted into the *GT(ROSA)26Sor* locus (hereafter *ROSA-Notch1^fl^*) [16]. Expression of this construct is suppressed by the floxed STOP cassette. After cre-mediated removal of the STOP cassette, the truncated cytoplasmic fragment encoded by the *Notch1* sequence causes constitutive Notch signaling activity. Mice with the floxed *Notch1^fl^* allele, Notch1*^tm2Rko/^*GridJ, have two *loxP* sites flanking exon 1 [17]. *Notch1* gain- or loss-of function conditional mutants with specific male germ cell activation or deletion of *Notch1* were produced by crossing with transgenic Tg(Stra8-icre)1Reb/J males (*Stra8-icre*). Initial experiments were conducted with Tg(Ddx4-cre)1Dcas/J (*Vasa-cre*), however all di-heterozygotes *ROSA-Notch1^fl^*, *Vasa-cre* died during embryonic development, suggesting non-testicular expression of cre transgene led to an activation of NICD in tissues/organs that was incompatible with the normal development. The *Stra8-icre* transgene expresses optimized icre recombinase under *Stra8* (stimulated by retinoic acid gene 8) genomic promoter fragment in the testis beginning from postnatal day 3 (P3) in early stage spermatogonia [18], [19]. Sibling *ROSA-Notch1^fl^*, *Stra8-icre* and *ROSA-Notch1^fl^*, + males and *Notch1^fl^*/*Notch1^fl^*, *Stra8-icre* and *Notch1^fl^*/*Notch1^fl^*, *+* males derived from the same crosses were used in all tests. Animal genotyping was performed using DNA isolated from ear pieces. Primers used for genotyping are listed in Table S1.

### Fertility and sperm count analysis

For fertility testing, individual mutant and control males were each mated continuously with two wild-type females for 2 months. The number of litters and litter sizes were compared. In each case four 2 month old mutant and three control males derived from the same litters were used. To assess sperm counts and motility, sperm was released from the cauda epididymis into M2 medium (Millipore, Billerica, MA), and scored under the microscope. Sperm DNA was extracted by DNeasy Kit (Qiagen, Valencia, CA).

### Histology, immunohistochemistry and apoptosis detection

The adult mouse organs were collected and fixed in 4% paraformaldehyde, washed with phosphate buffered saline (PBS), treated with a serial dilutions of ethanol, embedded in paraffin, and finally sectioned using standard protocols. The immunohistochemistry (IHC) was performed using the anti-NOTCH1 antibody (1∶500, Sigma-Aldrich, St. Louis, MO); protein detection was performed using a Vectastain ABC kit (Vector Laboratories, Burlingame, CA) as recommended. The color was developed with diaminobenzidine (DAB) as chromogen. Samples were counterstained with Harris Hematoxylin. Hematoxylin and eosin (H&E) staining was performed for routine analysis of the sections. A TUNEL assay was performed using an ApopTag Plus peroxidase in situ Apoptosis detection kit (Millipore, Billerica, MA). Stained slides were examined with a Carl Zeiss Axio A1 Microscope and images were captured by an AxioCam MRc5 CCD camera.

### Whole-mount immunofluorescence

The whole mounts of seminiferous tubules were prepared as described previously [20]. Mouse testes were decapsulated and cleaned by removing the interstitial cells and blood vessels in cold PBS. The dispersed seminiferous tubules were fixed in 4% paraformaldehyde overnight at 4°C. The tubules were washed in 0.01% Triton-PBS and transferred to microcentrifuge tubes where they were exposed to a series of dehydration and rehydration steps in PBS-diluted methanol and 0.1% Triton-PBS carried out at 4°C. Tubules were pre-incubated in PBS containing 0.1% Triton and 3% BSA for 2 hours at room temperature. After a rinse with 0.1% Triton-PBS, the tubules were then incubated with PLZF antibody (sc-22839, 1∶200, Santa Cruz Biotechnology, Santa Cruz, CA) for 1 hour at room temperature. Following primary antibody incubation, the tubules were incubated in fluorochrome secondary antibody (1∶1000) for 3 hours. A second one-hour blocking step was applied with PBS containing 0.1% Triton and 3% BSA at room temperature. Tubules were then rinsed with 0.01% Triton-PBS and incubated overnight with fluorochrome-conjugated anti-cleaved PARP (Asp 214) (BD Pharmingen, Franklin Lakes, NJ). The tubules were washed with 0.01% Triton-PBS six times for 15 minutes at 4°C, each time with agitation after exposure to primary and secondary antibodies. Specimens were analyzed with a Carl Zeiss Axio A1 Microscope and images were captured by an AxioCam MRc5 CCD camera.

### Flow Cytometry

Preparation of testicular cells for flow cytometry was performed as described before with some modifications [21], [22]. A was mixed by vortex in a 15-mL centrifuge tube. One decapsulated testis was treated with a solution containing 120 U/mL (0.5 mg/mL) of Collagenase Type IV (Sigma), 3 mL of Gey's Balanced Salt Solution (Sigma) and 10 µL of DNAse I (1 mg/mL stock solution in 50% Glycerol, Sigma), this was followed by vigorous shaking by hand until the seminiferous tubules started to dissociate. The tube was placed horizontally on a rocker for 15 minutes at 32°C. The supernatant was discarded and the tubules were washed twice in PBS for one min. The tubules were then incubated in a solution containing 5 µL of Trypsin, 10 µL of DNase, 3 mL of PBS and 1.5 µL of 2 M HCl for 15 min at 32°C. After incubation 400 µL of Fetal Bovine Serum (FBS) was added to the solution and the contents mixed gently using a disposable pipet with a wide orifice. This cell suspension was filtered using a 100 µm nylon cell strainer. The resulting monocellular filtrate was resuspended and centrifuged (1000 rpm at room temperature) for 4 minutes. A cell count was performed prior to the second wash. After washing, the resulting pellet was resuspended in BD Cytofix/Cytoperm™ (BD Biosciences, San Diego, CA) solution and incubated for 20 minutes at 4°C. After incubation in the above solution, a pellet was formed and the supernatant was discarded. The cells were washed with BD Perm/Wash™ buffer (10×10^6^ cells/mL), and aliquots consisting of 1×10^6^ cells per 100 µL reaction were pipetted into three 1.5 mL microcentrifuge tubes. Each respective sample was exposed to separate staining protocols with a) conjugated anti-cleaved PARP (Asp 214) (BD Pharmingen) (5 µL per 100 µL reaction overnight) followed by Propidium Iodide (10 µL per 100 µL reaction for 30 minutes); b) PLZF (Santa Cruz Biotechnology) (5 µL per 100 µL reaction for 30 minutes), followed by incubation with fluorochrome secondary antibody (10 µL per 100 µL reaction for 30 minutes), closing with incubation in Propidium Iodide; c) with conjugated anti-cleaved PARP (Asp 214), PLZF, and Propidium Iodide. After each staining step the cells were washed with 1 mL of BD Perm/Wash™ buffer. Once the staining process was completed for each sample, the cells were resuspended in 500 µLof BD Perm/Wash™ and analyzed by flow cytometry where 150,000 events/testis were recorded to generate the results. Additionally, gating was performed by excluding both 1N populations and Propidium Iodide background signal (varied among samples). Three 6 month old mutant and three sibling control males were used for analysis.

### RNA isolation and cDNA synthesis and real-time quantitative RT-PCR

Total RNA was isolated from mouse testis with Trizol (Invitrogen, Carlsbad, CA) according to the manufacturer's protocol. cDNA was synthesized using a Verso cDNA kit (Thermo Scientific, Waltham, MA) according to the manufacturer's protocol. The Go Taq Q-PCR master mix (Promega, Madison, WI) kit was used for the real time quantitative RT-PCR (QRT-PCR). Primers were designed to span a long exon to avoid amplification of genomic DNA; the primer sequences are shown in Table S2. The SybrGreen real time protocol was run on an Eppendorf Mastercycler ep realplex instrument (Eppendorf, Westbury, NY). The relative fold change in mRNA level was calculated by the comparative Ct (2^–ΔΔ^C_t_) method, where the β-actin (*Actb*) gene expression was used for normalization.

### Western blot analysis

SDS-PAGE and Western immunoblotting were performed using protein extracts isolated from the testis of mutant and control littermates of 140 day old animals. Following 4–15% gradient SDS-PAGE, the proteins were electro-transferred to a nitrocellulose membrane (Invitrogen). Rabbit anti-NOTCH1 antibody (Sigma) was used the primary antibody with the appropriate horseradish peroxidase-conjugated secondary antibody (Promega). Supersignal West Pico chemiluminescence kit (Thermo Scientific) was used to detect target proteins.

### Statistical analysis

Student *t-*test for two groups and one-way ANOVA for multiple group comparisons were used to assess significance of differences. Differences were expressed as mean ±SEM; P<0.05 was considered statistically significant. All analyses were performed using the GraphPad Software package (GraphPad Software, La Jolla, CA).

## Results

### Germ cell-specific NOTCH1 gain-of-function results in reduced male fertility

**Figure 1:**
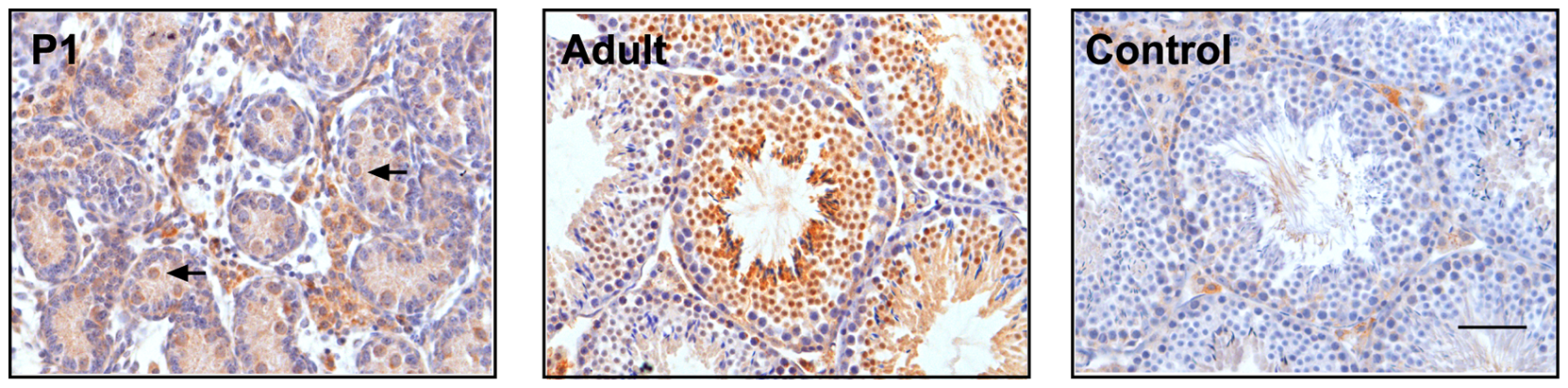
NOTCH1 expression in testis. Immunohistochemistry images of wild-type testis sections stained with anti-NOTCH antibodies. The staining was detected in pre-spermatogonial cells in P1 testis (arrows) and at later stages of spermatogenesis in adult (140 days old) testes. A weak non-specific staining was detected in interstitial testicular cells, but not in germ cells without primary antibody control sections. All images were acquired under the same magnification. The scale bar in control is 20 μm.

**Figure 2:**
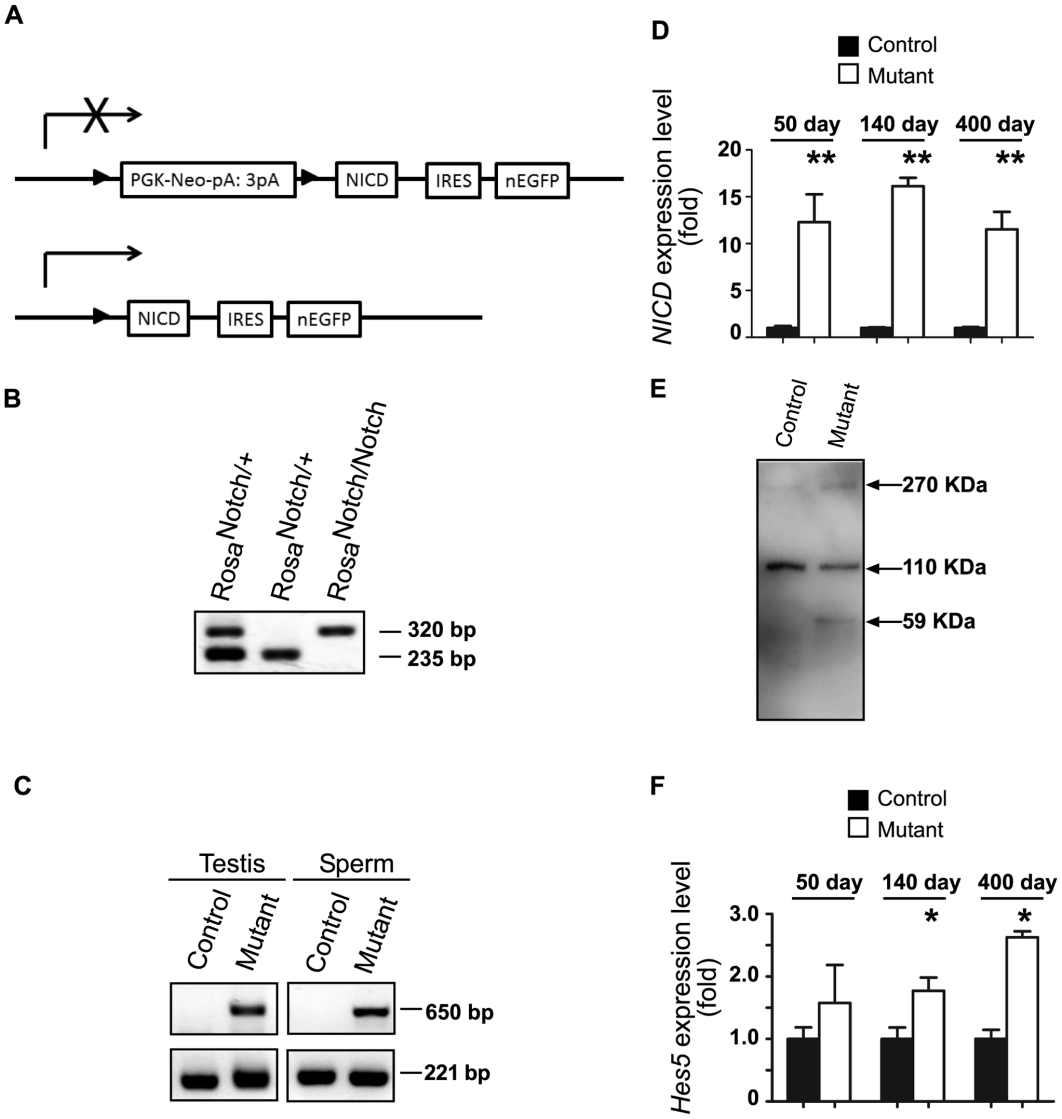
Germ cell-specific NOTCH1 gain-of-function in male mice. A. A transgene encoding an intracellular portion of the mouse *Notch1* gene (NICD) is inserted into the *ROSA* locus. The transcription of the transgene is disrupted by the presence of the floxed *Neo* cassette (PGK-Neo-pA:3pA). After cre-mediated removal of the *Neo* cassette, the transgene expresses the NICD. IRES, internal ribosome entry site, separates NICD-encoding sequence from nuclear enhanced green fluorescent protein (nEGFP) sequence. B. Genotyping of mice with *ROSA-Notch1^fl^* allele (320 bp) and wild-type allele (235 bp) using ear DNA PCR. C. Detection of activated *ROSA-Notch1* allele in genomic DNA isolated from mutant (*ROSA-Notch1^fl^/+, Stra8-icre/+*) adult testes and epidydimal sperm but not control (*ROSA-Notch1^fl^/+, +*) DNA. The 650 bp fragment is specific for the activated *ROSA-Notch1* allele. Control PCR with primers specific for *Ctnnb1* locus was used to confirm the quality of isolated DNA. D. The *NICD* expression was increased in mutant testis. The QRT-PCR analysis of *NICD* expression in RNA isolated from control (n = 4, 3, and 3, respectively) and gain-of-function NOTCH1 mutants (n = 4, 4, and 3, respectively) at day 50, 140, and 400 after birth. The data are normalized to the expression of *Actb* gene and the level of gene expression in control samples was assumed to be equal 1 at each time point. *P<0.05. E. Expression of NICD protein in mutant testis. Western blot analysis was used to verify transgenic NICD expression in testicular lysates of adult mutant and normal males. The transgenic NICD (MW  = 58.69 kDa) is expressed only in mutant. f. Increased *Hes5* gene expression in mutant testis. The QRT-PCR analysis of *Hes5* expression in RNA isolated from control (n = 4, 3, and 3, respectively) and gain-of-function NOTCH1 mutants (n = 4, 4, and 3, respectively) at day 50, 140, and 400 after birth. The data are normalized to the expression of *Actb* gene and the level of gene expression in control samples was assumed to be equal 1 at each time point. *P<0.05.

First, we used IHC to check the localization of NOTCH1 in the mouse testis. Positive staining was found in spermatogonial cells in the neonatal testis; in adult males the strongest staining was detected in differentiating spermatocytes and spermatids (Figure 1). To analyze the effect of constitutive activation of NOTCH1 on male germ cells we used a NICD transgene with a STOP cassette flanked by loxP sites. The expression of the *Stra8-icre* transgene in double transgenic males caused the removal of the STOP cassette and constitutive expression of NICD in cells where the transgene was first expressed, as well as in any of their progenitor cells (Figure 2A). It was previously shown that the chimeric expression of the transgene begins as early as at P3 in early spermatogonial cells and gradually increases with the progression of germ cell differentiation [18], [19]. PCR analysis of testicular genomic DNA and DNA isolated from epididymal sperm confirmed the efficient removal of the STOP cassette (Figure 2C). QRT-PCR analysis of testis RNA using primers derived from the region of *Notch1* mRNA encoding NICD, showed an activation of transgene expression (Figure 2D). Western blot analysis of the testicular samples with anti-NOTCH1 antibody revealed the presence of an additional band in mutant samples with a molecular weight corresponding to the NICD derived from the transgene (Figure 2E). One of the primary downstream NOTCH1 target genes, *Hes5*, was significant upregulated in mutant testis (Figure 2F), suggesting functional activation of the canonical NOTCH effector. The expression level of other members of the NOTCH pathway (*Notch2*, *Notch3*, *Notch4*, *Numb, Jag1*, *Dll1*, *Dll4, Hes1)* did not change in mutant testis (Figure S1).

**Figure 3:**
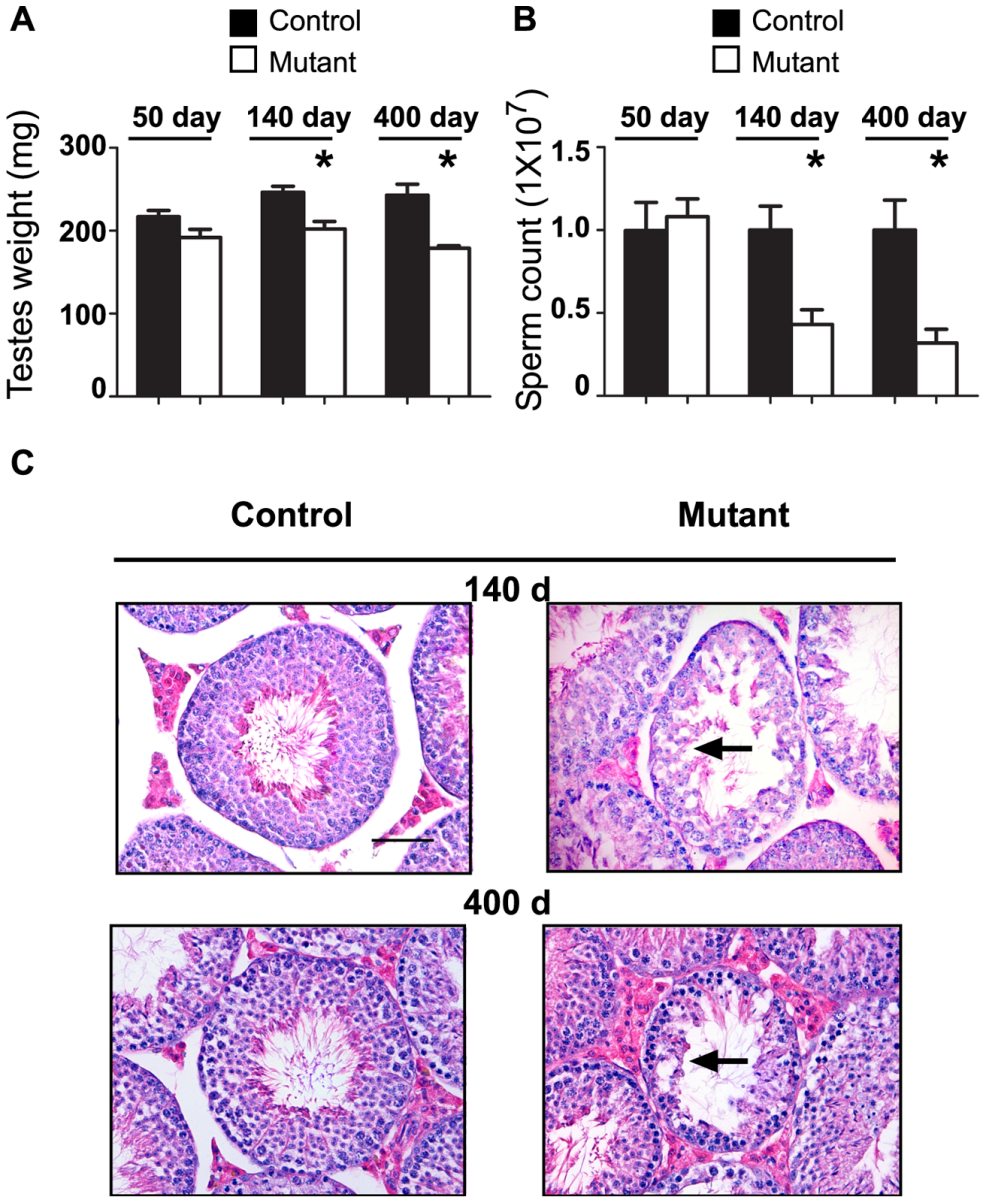
Abnormal spermatogenesis in NOTCH1 gain-of-function mice. A. Decrease in the testes weight in 50-, 140-, and 400-day-old mutant vs littermate control males. n≥3 in each group. *P<0.05. B. Age-dependent decrease in epididymal sperm counts in mutant males. The same animals as in A were used. *P<0.05. C. Normal spermatogenesis in control testes of different age (Stage 8) and corresponding sections from mutant animals. Note abnormal spermatogenesis (arrows) with no germ cells beyond spermatocyte stage and the distorted integrity of seminiferous tubules.

Analysis of mutant testes' weight and epididymal sperm count revealed a considerable decrease with age (Figure 3A, B). While at 50 days no significant differences were detected at 140 and 400 days, the mutant testes' weight and sperm counts were significantly reduced. No significant variation among control and mutant groups in seminal vesicle and epididymis weight was detected (data not shown). Histological evaluation of the testicular sections of 50-day-old mutants did not reveal any abnormalities in spermatogenesis. The testes of 140-day-old mutant mice displayed morphological distortions of spermatogenesis in some of the seminiferous tubules (Figure 3C). In 400-day-old mutant testes abnormal tubules seem to deteriorate significantly with only spermatogonial cells present at the basement membrane (Figure 3C).

A high mortality rate was previously recorded in mice with a ubiquitously activated NICD transgene during early prenatal development at embryonic day 10.5–11.5 (E10.5–11.5) [23]. Litter size of heterozygous *ROSA-Notch1^fl^/*+, *Stra8-icre* was reduced about 50% compared to wild-type littermate control males. We tested all progeny from the mutant males for the presence of activated NICD or floxed *ROSA-Notch1^fl^* alleles. None of the more than 40 live pups analyzed born from *ROSA-Notch1^fl^/*+, *Stra8-icre* males had the mutant alleles; they all had +/+ wild-type genotype with or without the *Stra8-icre* transgene. These data suggest a 100% efficiency of cre-mediated removal of STOP cassette in germ cells resulting in embryonic lethality of the progeny with a mutant allele.

**Figure 4:**
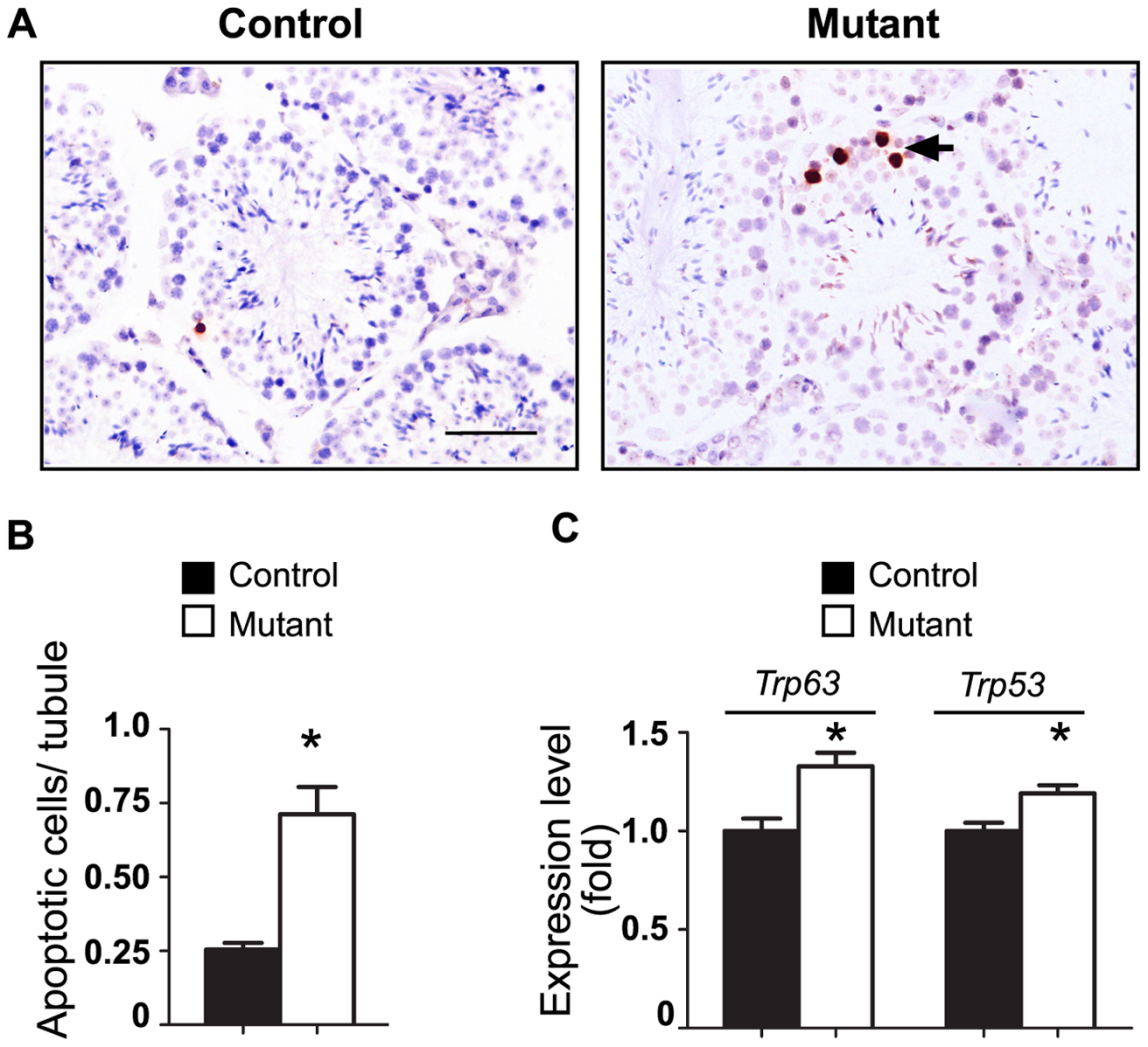
Increased germ cell apoptosis in seminiferous tubules of NOTCH1 gain-of-function mice. A. Representative testicular sections of adult testes. Arrows show the dark-stained apoptotic cells located at the basement membrane of the tubes. B. The average number of apoptotic cells per tubules in mutant testes is significantly increased (*P<0.05). C. Relative expression of pro-apoptotic genes increases in mutant testis (*P<0.05). QRT-PCR results were normalized to the *Actb* gene expression. The expression of *Trp53* and *Trp63* genes in control samples was assumed to be equal to 1. Number of samples n≥3 in each group.

QRT-PCR analysis of proliferation markers PCNA and Ki67 did not reveal significant variability of expression in mutant testes with respect to the control mice (data not shown). IHC with Ki67 supported these results with no differences detected between mutant and wild-type testis (data not shown). Conversely, the analysis of cell apoptosis in mutant testis revealed a significant increase in germ cells apoptosis (Figure 4). In mutant testis, apoptosis was restricted to cells in close proximity to the basement membrane of seminiferous tubules, suggesting that the activation of NOTCH1 signaling led to apoptosis in spermatogonial cells (Figure 4A, B). QRT-PCR analysis of *Bax*, *Bcl2*, *Fas*, *Fasl*, *Nfkb1*, *Trp53*, and *Trp63* genes revealed elevated expression levels of the pro-apoptotic markers *Trp53*, *Trp63* in mutant testis (Figure 3C, S2). No change in the expression of Sertoli or Leydig cell specific markers (*Amh*, *Gdnf*, *Kitl*, or *Star*) (Figure S2) was detected in the mutant testis, suggesting that the activation of NOTCH signaling in germ cells did not affect the functions of testicular somatic cells.

### Increased apoptosis of spermatogonial cells in mutants with activated NOTCH1 signaling

**Figure 5:**
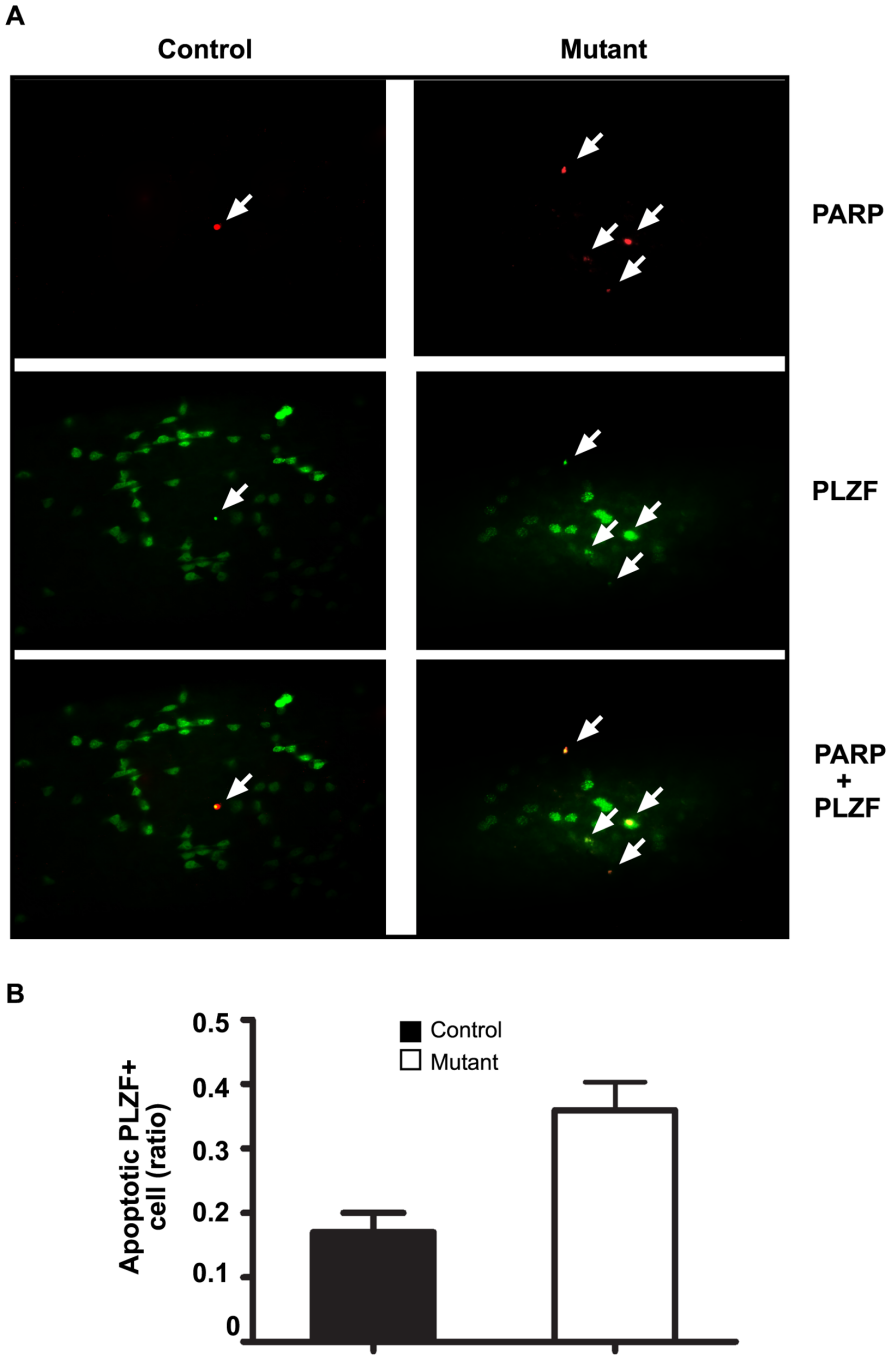
Increased cell apoptosis in spermatogonial cells of NOTCH1 gain-of-function males. A. The majority of cleaved PARP-positive cells were also PLZF-positive. Representative images of PARP (Red, arrows) and PLZF (Green) double-immunofluorescent staining of the whole-mount seminiferous tubules from control (*ROSA-Notch1^fl^/+*) and mutant (*ROSA-Notch1^fl^/+, Stra8-icre/+*) testes. Double staining in merged images is yellow. B. The results of flow cytometry analysis with the cleaved PARP- and PLZF-labeled testicular cells isolated from 3 control and 3 mutant 6 month old males. The proportion of PLZF-positive spermatogonial cells entering apoptosis was significantly increased in mutants (P>0.05).

The close proximity of apoptotic germ cells to the basement membrane of seminiferous tubules in mutant males indicated that the affected cells might be spermatogonia. The whole-mount double immunofluorescent staining with PLZF, a marker of spermatogonial cells, and PARP cleavage fraction, a marker of apoptosis, showed the overlapping pattern in the majority of cells (Figure 5A). Quantitative analysis of such double staining was performed using flow cytometry (Figure 5B). About two times more (P<0.05) PLZF-positive spermatogonial cells were undergoing apoptosis in males with activated NOTCH1 signaling than PLZF-positive cells in littermate control testes.

### Expression of *Neurog3* is significantly decreased in testis with activated NOTCH1 signaling

**Figure 6:**
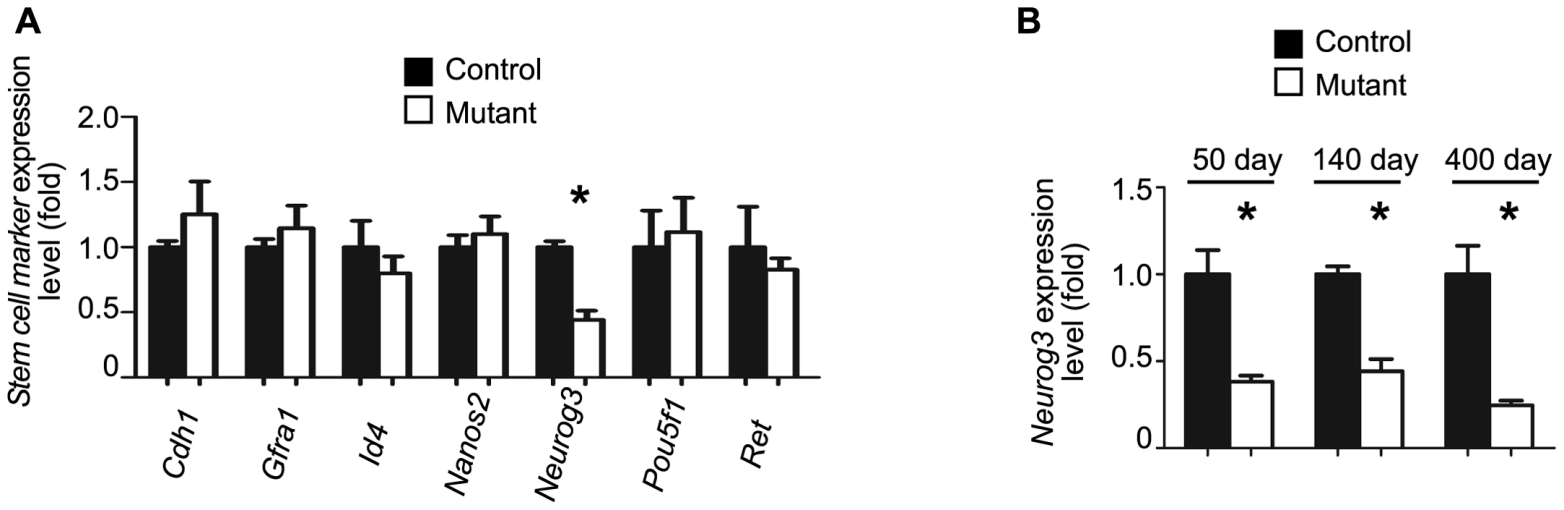
Expression of spermatogonial stem cell marker Neurog3 decreases in mutant testis. A. Expression of spermatogonial stem cell markers in control and NOTCH1 gain-of-function mutants. The expression of SSC markers in adult (140 day) control and mutant testes were compared by QRT-PCR. The *Neurog3* gene is significantly down-regulated in mutant testis (*P<0.05). QRT-PCR results were normalized to the *Plzf* gene expression. The expression of each gene in control samples was assumed to be equal to 1. Number of samples N≥3 in each group. B. The expression of *Neurog3* gene is reduced in mutant testes of different ages. *P<0.05. Number of samples N≥3 in each group. QRT-PCR results were normalized to *Plzf* gene expression.

Spermatogonial cells are the most complex cell population comprising undifferentiated SSCs and cells entering the differentiation pathway. To identify the germ cell population affected by apoptosis and possible molecular targets of NICD overexpression we used QRT-PCR to analyze the expression of markers specific for different spermatogonial cells. Initially, we checked early stage spermatogonial stem cell markers, including *Cdh*, *Graf*, *Id4*, *Nanos2*, *Neurog3*, *Pou5f1*, and *Ret* (Figure 6A) in 140 day old testis. When normalized to the expression of *Plzf*, or to ubiquitously expressed β-actin (*Actb*), only *Neurog3* expression was significantly decreased in mutants (Figure 6A, S3). Interestingly, this difference was significant even in 50 day old males and it was further increased in older, 400 day old mice. We then checked the expression of genes associated with differentiating spermatogonia cells, including *Dmrt1*, *Kit*, *Lin28a*, *Sohlh1*, *Sohlh2*, *Sox3*, *Stra8*; none of these markers showed significant changes (Figure S3). Thus, the activation of NOTCH signaling in spermatogonia cells resulted in the reduction of *Neurog3* expression.

### Conditional deletion of Notch1 in germ cells has no effect on spermatogenesis and male fertility

Using the same *Stra8-icre* transgene, we produced mice with a conditional deletion of the floxed *Notch1^fl^* allele in spermatogonial cells. Analysis of mutants at different stages of development revealed no visible phenotypic abnormalities. The weight of the testis, epididymis, and seminal vesicle was comparable to those of the control. Additionally, we detected no significant change in sperm count or motility in mutants (Table 1). Litter size was not altered in comparison to wild-type littermates (Table 1). No histological defects were detected in testis sections from *Notch1^fl^/Notch1^fl^*, *Star8-icre* males (Figure S4).

**Table 1:** Characterization of reproductive phenotype of male mice with conditional deletion of *Notch1* in germ cells.

	Testes weight (mg)	Epididymis weight (mg)	Seminal vesicle weight (mg)	Sperm (×10^6^)	Litter size
Control (n = 3)	213.2±7.2	87±2.6	317.1±42.4	15.9±2.1	15.3±1.3
Mutant (n = 3)	211.7±16.1	85±5.4	367.4±60.3	19.1±1.6	15±0.3

## Discussion

The role of NOTCH signaling in germ cell differentiation is well established in *C. Elegans* and Drosophila. Based on the expression of NOTCH and members of the canonical NOTCH pathway in the testis, it was suggested that NOTCH signaling may be important for germ cell differentiation [24], [25]. While different NOTCH receptors were shown to be expressed in the mammalian testis, including germ cells, their role in spermatogenesis remained unclear. Recently, using a gene loss- and gain-of-function approach it was shown that NOTCH signaling in Sertoli cells plays a role in accelerating gonocyte exit from quiescence [5], [26]. The question remained however whether NOTCH signaling has any role in germ cell differentiation. The direct gene targeting of *Notch1* gene or *Pofut1* in germ cells using conditional deletion of floxed alleles with *Stra8-icre*, showed that those genes are dispensable for normal spermatogenesis [15]. However, due to a redundancy of NOTCH receptors expressed in germ cells, the deletion of *Notch1* might be compensated by other members of this family. Similarly, POFUT1 is not the only factor affecting NOTCH folding and cell surface expression in mammals [27]. As reported here, we also showed that conditional germ cell-specific ablation of the *Notch1* gene is dispensable for normal spermatogenesis. Alternatively, our data showed that the constitutive activation of NOTCH signaling in germ cells causes progressive decrease in sperm production and testis weight.

Previously, it was reported that overexpression of NICD driven by an MMTV promoter in mice resulted in compete male sterility [28]. While sterility was primarily attributed to the abnormal development of the reproductive tract, and specifically ectopic growth and blockage of efferent ducts and epididymal hyperplasia, the testicular sections revealed the presence of some abnormal tubules devoid of spermatids. The MMTV promoter is expressed in male germ cells, thus the results of the MMTV-NICD transgene somewhat mirror the results obtained in our experiments.

The expression analysis of NOTCH1 using IHC, confirmed previously reported pattern. Some positive staining was observed in early neonatal germ cells, suggesting NOTCH1 expression in spermatogonial cells. In the adult testis, much stronger staining was detected in spermatocytes and spermatids. As noted, the deletion of the *Notch* gene in germ cells did not affect spermatogenesis while overexpression did. Thus, the first question arises as to whether the detected pattern of NOTCH1 expression correlates with the effects of NICD overexpression on germ cells. In addition, what stage of spermatogenesis is most likely affected by disregulation of NOTCH signaling in *ROSA-Notch1^fl^/*+, *Stra8-icre* males? To answer these questions we first reviewed the reported pattern of *Stra8-icre* expression and hence the cells where NICD could be activated.

Cre expression in the *Stra8-icre* transgenic testis was first detected on postnatal day 3 [18]. Thus, activation of NICD should occur in very early stages of germ cell differentiation, in nascent spermatogonial progenitor cells. The activated transgene will thereafter be transmitted to all cells derived from such spermatogonia. The question then arises, why did such abnormal signaling not disrupt spermatogenesis completely if it happened so early during spermatogenesis? One explanation might be related to the incomplete or chimeric expression of the *Stra8-icre* transgene in the early spermatogonial cell population. Indeed, it was shown that while the expression of cre was detectable in the postnatal testis, only a subset of PLZF-positive spermatogonial cells expressed cre, whereas in other cells, cre was not detected [19]. The expression of *Stra8-icre* progressively increases in later stages of spermatogenesis especially in premeiotic spermatocytes [18], [19], which in our experiments should result in germ cells with activated NICD. Eventually, as the data indicate here, in all sperm with the mutant allele, there was a removal of the floxed STOP cassette. Such sperm was apparently fully functional giving rise to the expected 50% of the embryos. Based on these data we can assume that the activation of NICD had no effect on pre- and post-meiotic spermatocytes, spermatids, or spermatozoa. This conclusion correlates with the observed increase of endogenous NOTCH1 expression in differentiating pre- and post-meiotic germ cells in wild-type testis. Thus, only spermatogonial cells would be sensitive to activated NOTCH signaling. The incomplete expression of cre at these early germ cells results therefore in NICD transgene silence in significant number of spermatogonia and their normal differentiation.

Not surprisingly, no notable change in testicular histological appearance or sperm counts was detected in young mutant males. The first wave of spermatogenesis originated directly from gonocytes [29] in which NOTCH was not yet activated. As the mutant males aged, the spermatogenic defects manifested more clearly with a simultaneous decrease in testes weights and sperm counts. Thus, the pool of spermatogonial cells with activated NICD increased with time most probably due to the accumulation of mutated cells within the SSC population.

What is the mechanism of decreased sperm production in the NICD overexpressing testis? We have shown that the activation of NOTCH1 signaling in male germ cells correlated with overexpression of pro-apoptotic cell markers, Trp53 and Trp63. The germ cell apoptosis was further confirmed by the direct count of apoptotic cells in seminiferous tubules. Significantly, most of the apoptotic cells were located close to the basement membrane, suggesting that spermatogonia were primarily affected. Indeed, the flow cytometry and the double immunostaining with PLZF and pro-apoptotic marker cleaved PARP showed that the apoptosis was increased in PLZF-positive cells. However, the relatively modest increase in apoptosis observed in mutant testes indicated there could be other mechanisms inhibiting sperm production. Analysis of all but one germ cell stage-specific markers did not reveal any differences in their expression. The only significantly down-regulated gene affected by NOTCH1 signaling was *Neurog3*. This marker of the SSC subpopulation is expressed very early, mainly in undifferentiated and early differentiating spermatogonia [30]. While there is an overlap, NEUROG3 is predominantly expressed in GFRA1-negative cells, which are likely to represent transit-amplifying spermatogonia [6], [31]–[33]. The function of NEUROG3 in SSCs is not quite clear but in other types of stem cells, such as pancreatic stem cells, this factor controls cell entry into the differentiation pathway [34]. It was demonstrated that such a transition from high to intermediate NOTCH activity was accompanied with the downregulation of HES1, the transcriptional suppressor of NEUROG3. This resulted in the de-repression of *Neurog3* and an initiation of endocrine cell differentiation [34]. In our mutants, we detected an increase in the expression of another member of the Hairy and enhancer of split gene family, *Hes5,* also an established downstream target of NOTCH1 signaling. Moreover, the analysis of the promoter region of mouse *Neurog3* gene revealed the presence of two conservative HES5 N-box binding sites (CACNAG) at positions −3949 bp and −4662 bp from the start codon. While we have not performed direct promoter binding or activation studies, one can speculate that *Neurog3* gene expression might be regulated by HES5.

**Figure 7:**
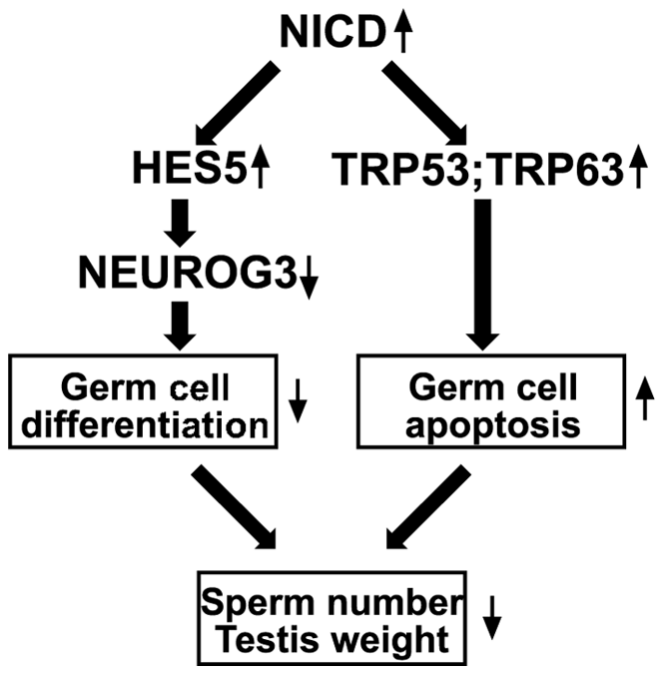
The effect of transgenic activation of NOTCH1 signaling in spermatogonial germ cells. In male spermatogonial cells, the known target of NOTCH1, HES5 is up-regulated and causes a decrease of NEUROG3 expression. This leads to a failure of spermatogonial cell differentiation. Simultaneously, the activation of NOTCH1 signaling induces an increased apoptosis with overexpression of pro-apoptotic genes TRP53 and TRP63. Both pathways contribute to the progressive decrease in sperm number and testis weight.

Based on our data we propose the following scenario to explain the age-dependent decrease in sperm production in *ROSA-Notch1^fl^/*+, *Stra8-icre* males. The activation of NOTCH1 in SSCs results in the increased expression of HES5. Subsequently, it results in partial NEUROG3 suppression and a failure of SSC differentiation. Simultaneously, the NOTCH1 signaling results in an activation of pro-apoptotic genes TRP53 and TRP63, triggering spermatogonial cell apoptosis (Figure 7). Both events lead to the progressive age-dependent reduction of germ cell number including sperm in the mutants. SSCs pool remains however less unaffected as the majority of these cells do not have an activated NICD. Taken together, our findings suggest that the control of NOTCH1 signaling is required for initiation of SSC differentiation.

## Supporting Information

Figure S1 **Analysis of NOTCH signaling pathway related genes in testis with constitutive expression of NICD**. The QRT-PCR analysis of genes' expression in RNA isolated from control (n =  3) and gain-of-function NOTCH1 mutants (n = 4) at day 140 after birth. The data are normalized to the expression of *Actb* gene and the level of gene expression in control samples was assumed to be equal 1. No statistically significant difference was detected.
(TIF)

Figure S2 **Analysis of apoptosis related and testicular somatic specific genes in testis with constitutive expression of NICD**. The QRT-PCR analysis of genes' expression in RNA isolated from control (n = 3) and gain-of-function NOTCH1 mutants (n = 4) at day 140 after birth. The data are normalized to the expression of *Actb* gene and the level of gene expression in control samples was assumed to be equal 1. No statistically significant difference was detected.
(TIF)

Figure S3 **Analysis of spermatogonial cell related genes in testis with constitutive expression of NICD**. The QRT-PCR analysis of genes' expression in RNA isolated from control (n = 3) and gain-of-function NOTCH1 mutants (n = 4) at day 140 after birth. The data are normalized to the expression of *Plzf* gene and the level of gene expression in control samples was assumed to be equal 1. No statistically significant difference was detected.
(TIF)

Figure S4 **Normal spermatogenesis in males with conditional inactivation of** ***Notch1*** **gene in germ cells**. H&E staining of adult 140 day old wild-type (control) and *Notchfl/Notchfl Stra8-icre* (Mutant) testes.
(TIF)

Table S1 **PCR primers used for genotyping.** For identification of different *ROSA-Notch1* alleles mutant, three primers are used: RosaWtR; RosaKoR and RosaF. The wild-type (WT) allele produced a 235 bp PCR fragment and the floxed allele produced a 320 bp fragment. RosadelF and RosadelR primers were used to verify the deletion of floxed STOP cassette: successful recombination was detected by the presence of 650 bp PCR fragment. The *Notch^fl^* mutant and wild-type alleles were identified by NotchF and NotchR primers. The *Notch^fl^* allele produced a 281 bp PCR product and the wild-type allele produced a 231 bp PCR product. The iCreF and iCreR primers were used to detect *Stra8-icre* transgene and produced a 164 bp PCR product.
(DOC)

Table S2 **QRT-PCR Primers.**
(DOC)
