# Supplementary figures and images for: NOTCH1 Gain of Function in Germ Cells Causes Failure of Spermatogenesis in Male Mice
Source: PLoS One. 2013 Jul 30;8(7):e71213. doi: 10.1371/journal.pone.0071213 (PMC3728026; doi:10.1371/journal.pone.0071213)

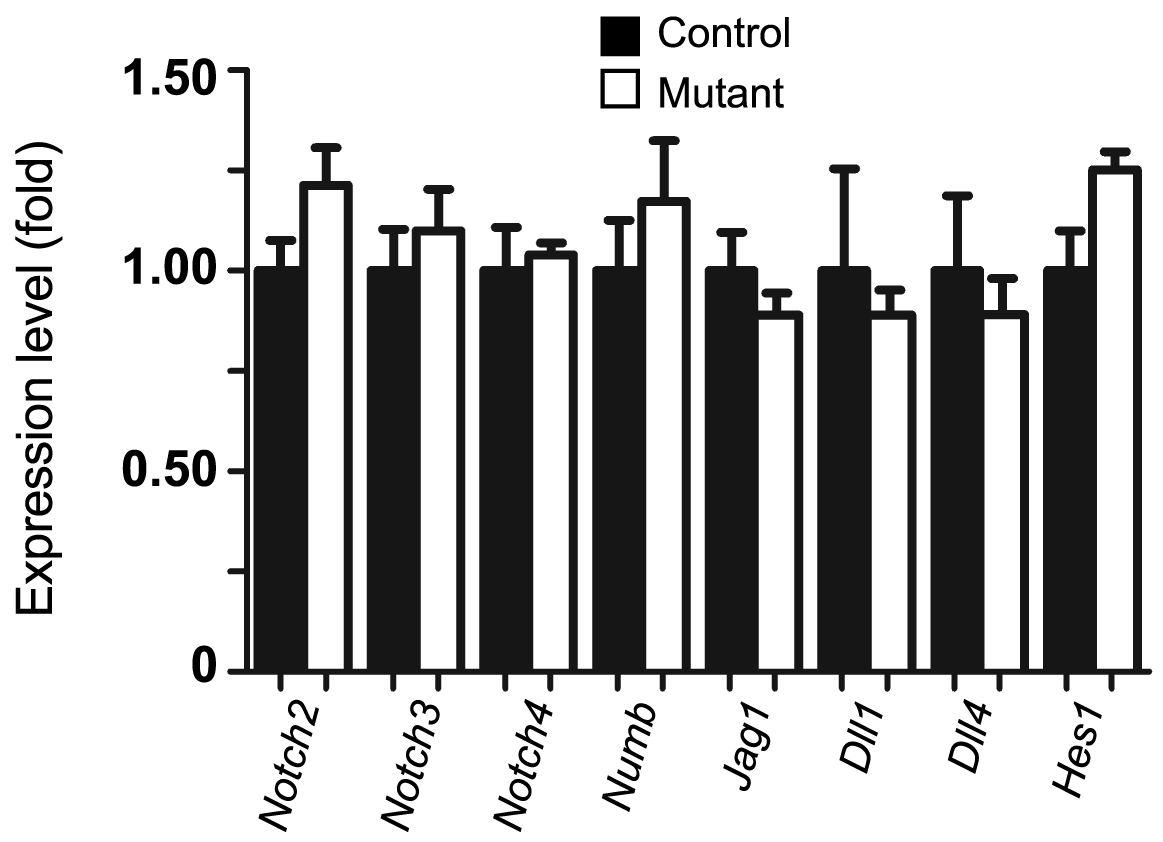

Supplement: Figure S1 — Analysis of NOTCH signaling pathway related genes in testis with constitutive expression of NICD. The QRT-PCR analysis of genes' expression in RNA isolated from control (n = 3) and gain-of-function NOTCH1 mutants (n = 4) at day 140 after birth. The data are normalized to the expression of Actb gene and the level of gene expression in control samples was assumed to be equal 1. No statistically significant difference was detected. (TIF) [file pone.0071213.s001.tif]

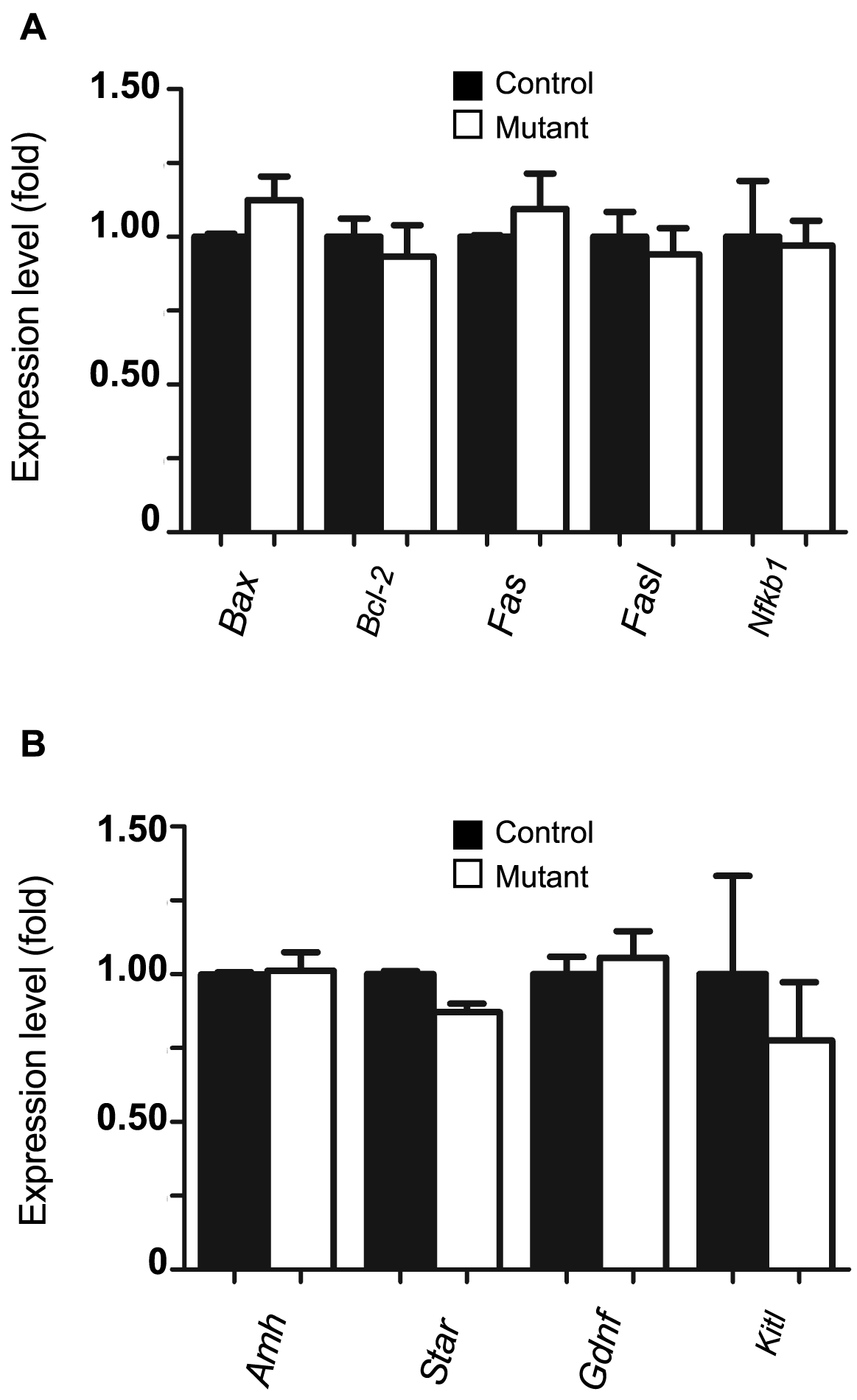

Supplement: Figure S2 — Analysis of apoptosis related and testicular somatic specific genes in testis with constitutive expression of NICD. The QRT-PCR analysis of genes' expression in RNA isolated from control (n = 3) and gain-of-function NOTCH1 mutants (n = 4) at day 140 after birth. The data are normalized to the expression of Actb gene and the level of gene expression in control samples was assumed to be equal 1. No statistically significant difference was detected. (TIF) [file pone.0071213.s002.tif]

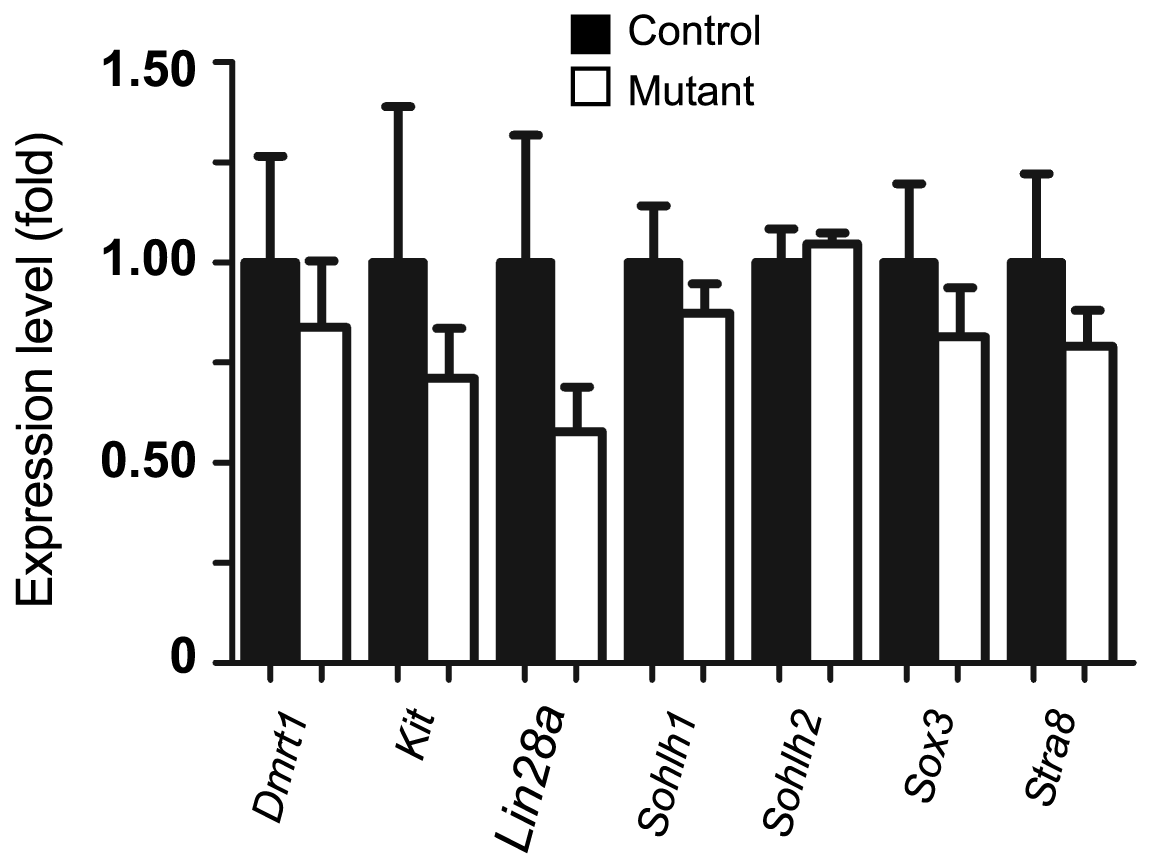

Supplement: Figure S3 — Analysis of spermatogonial cell related genes in testis with constitutive expression of NICD. The QRT-PCR analysis of genes' expression in RNA isolated from control (n = 3) and gain-of-function NOTCH1 mutants (n = 4) at day 140 after birth. The data are normalized to the expression of Plzf gene and the level of gene expression in control samples was assumed to be equal 1. No statistically significant difference was detected. (TIF) [file pone.0071213.s003.tif]

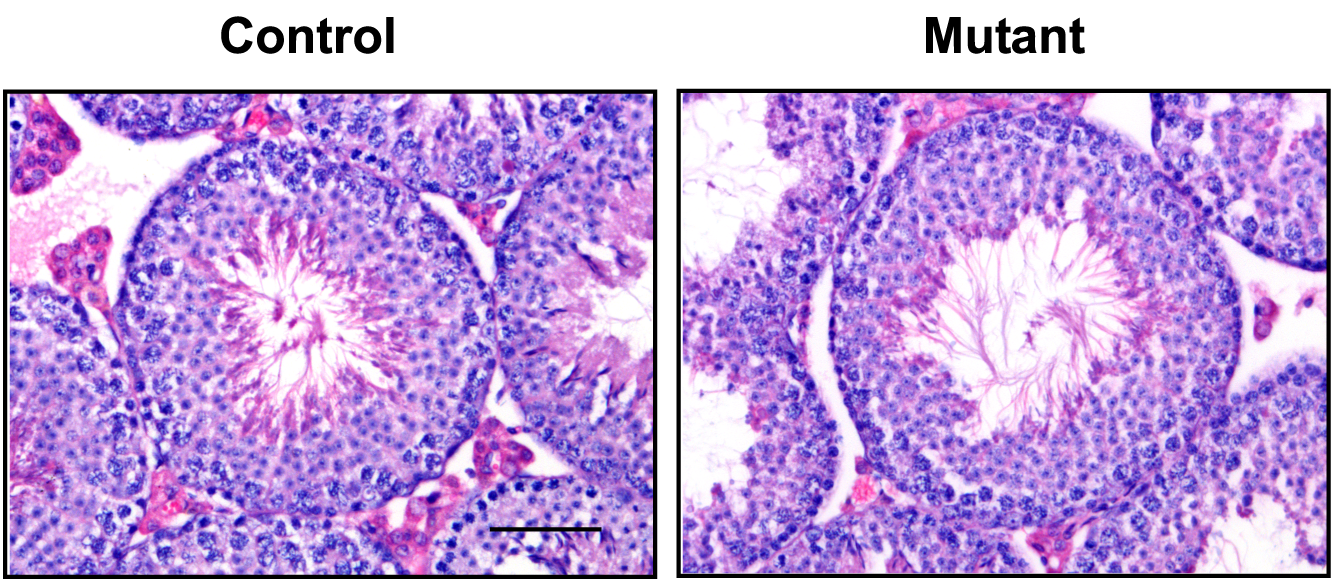

Supplement: Figure S4 — Normal spermatogenesis in males with conditional inactivation of Notch1 gene in germ cells. H&E staining of adult 140 day old wild-type (control) and Notchfl/Notchfl Stra8-icre (Mutant) testes. (TIF) [file pone.0071213.s004.tif]
