# Supplementary material for: NOTCH1 Gain of Function in Germ Cells Causes Failure of Spermatogenesis in Male Mice
Source: PLoS One. 2013 Jul 30;8(7):e71213. doi: 10.1371/journal.pone.0071213 (PMC3728026; doi:10.1371/journal.pone.0071213)
Supplement: Table S1 — PCR primers used for genotyping. For identification of different ROSA-Notch1 alleles mutant, three primers are used: RosaWtR; RosaKoR and RosaF. The wild-type (WT) allele produced a 235 bp PCR fragment and the floxed allele produced a 320 bp fragment. RosadelF and RosadelR primers were used to verify the deletion of floxed STOP cassette: successful recombination was detected by the presence of 650 bp PCR fragment. The Notchfl mutant and wild-type alleles were identified by NotchF and NotchR primers. The Notchfl allele produced a 281 bp PCR product and the wild-type allele produced a 231 bp PCR product. The iCreF and iCreR primers were used to detect Stra8-icre transgene and produced a 164 bp PCR product. (DOC) [file pone.0071213.s005.doc]

**Table S1. PCR primers used for genotyping.** For identification of different *ROSA-Notch1* alleles mutant, three primers are used: RosaWtR; RosaKoR and RosaF. The wild-type (WT) allele produced a 235 bp PCR fragment and the floxed allele produced a 320 bp fragment. RosadelF and RosadelR primers were used to verify the deletion of floxed STOP cassette: successful recombination was detected by the presence of 650 bp PCR fragment. The *Notch^fl^* mutant and wild-type alleles were identified by NotchF and NotchR primers. The *Notch^fl^* allele produced a 281 bp PCR product and the wild-type allele produced a 231 bp PCR product. The iCreF and iCreR primers were used to detect *Stra8-icre* transgene and produced a 164 bp PCR product.

| **Primer Name** | **Primer sequence** | **Product size (bp)** |
| --- | --- | --- |
| RosaF | AAAGTCGCTCTGAGTTGTTAT | 235 (WT)  320 (*ROSA-Notch1^fl^*) |
| RosaWtR | TAAGCCTGCCCAGAAGACTC |
| RosaKoR | GAAAGACCGCGAAGAGTTTG |
| RosadelF | AAGGGAGCTGCAGTGGAGTA | 650 |
| RosadelR | CTTCTTCTTGCTGGCCTCTG |
| iCreF | GGAGAATCAGAAAGGAGAATGTG | 164 |
| iCreR | GCAGCAGGGTGTTGTAGG |
| NotchF | TGCCCTTTCCTTAAAAGTGG | 231 (WT)  281 (*Notch^fl^*) |
| NotchR | GCCTACTCCGACACCCAATA |
