# Supplementary material for: NOTCH1 Gain of Function in Germ Cells Causes Failure of Spermatogenesis in Male Mice
Source: PLoS One. 2013 Jul 30;8(7):e71213. doi: 10.1371/journal.pone.0071213 (PMC3728026; doi:10.1371/journal.pone.0071213)
Supplement: Table S2 — QRT-PCR Primers. (DOC) [file pone.0071213.s006.doc]

**Table S2. QRT-PCR Primers.**

| **Target gene** | **Forward** | **Reverse** |
| --- | --- | --- |
| *Actb* | CTAAGGCCAACCGTGAAAAG | ACCAGAGGCATACAGGGACA |
| *Amh* | AGCTGGACACCATGCCTTTC | AGGGTCTCTAGGAAGGGGTC |
| *Bax* | GTGAGCGGCTGCTTGTCT | GGTCCCGAAGTAGGAGAGGA |
| *Bcl2* | GTACCTGAACCGGCATCTG | GGGGCCATATAGTTCCACAA |
| *Cdh1* | ATCCTCGCCCTGCTGATT | ACCACCGTTCTCCTCCGTA |
| *Dll1* | TTCAACTGTGAGAAGAAGATGGAT | GCCGAGGTCCACACACTT |
| *Dll4* | AGGTGCCACTTCGGTTACAC | GGGAGAGCAAATGGCTGATA |
| *Dmrt1* | AGAAGCCAAAGCCAGTGTGT | AGGGAGACCAAGCCAGAATC |
| *Fas* | TGCAGACATGCTGTGGATCT | CTTAACTGTGAGCCAGCAAGC |
| *Fasl* | ACCGGTGGTATTTTTCATGG | AGGCTTTGGTTGGTGAACTC |
| *Gdnf* | TCCAACTGGGGGTCTACG | GACATCCCATAACTTCATCTTAGAGTC |
| *Gfrα1* | ACTCCTGGATTTGCTGATGTCGG | CGCTGCGGCACTCATCCTT |
| *Hes1* | ACACCGGACAAACCAAAGAC | CGCCTCTTCTCCATGATAGG |
| *Hes5* | GATGCTCAGTCCCAAGGAGA | AGCTTCAGCTGCTCTATGCTG |
| *Id4* | AGGGTGACAGCATTCTCTGC | CCGGTGGCTTGTTTCTCTTA |
| *Jag1* | GAGGCGTCCTCTGAAAAACA | ACCCAAGCCACTGTTAAGACA |
| *Kit* | AGATCTCGGACAGCACCAAG | GAGTTGACCCTCACGGAATG |
| *Kitl* | AGCGCTGCCTTTCCTTATG | CGCAGATCTCCTTGGTTTTG |
| *Lin28* | ACATGCAGAAGCGAAGATCC | CCTTGGCATGATGGTCTAGC |
| *Nanos2* | GACCATCCATCTATCTTCACCT | CCTCCTCTAGTTCCTGTAACC |
| *Neurog3* | GCTATCCACTGCTGCTTGA | CCGGGAAAAGGTTGTTGTGT |
| *NICD* | GGACATGCAGAACAACAAGG | CAGTCTCATAGCTGCCCTCA |
| *Notch2* | TGCCTGTTTGACAACTTTGAGT | GTGGTCTGCACAGTATTTGTCAT |
| *Notch3* | AGCTGGGTCCTGAGGTGAT | AGACAGAGCCGGTTGTCAAT |
| *Notch4* | GGACCTGCTTGCAACCTTC | CCTCACAGAGCCTCCCTTC |
| *Nfkb1* | CACTGCTCAGGTCCACTGTC | CTGTCACTATCCCGGAGTTCA |
| *Numb* | CCACATCAGTGGCAGACAGA | TTCTACGTGGCCGAGGTACT |
| *Ztb* | AATGCATTTACTGGCTCATTCA | CAGGGCATCCTCCTTTGAG |
| *Pou5f1* | GTTGGAGAAGGTGGAACCAA | CTCCTTCTGCAGGGCTTTC |
| *Ret* | TCCCTTCCACATGGATTGA | ATCGGCTCTCGTGAGTGGTA |
| *Sox3* | CGCTGGCTTCTGACCACT | GCAAACACCACAGCGATTC |
| *Sohlh1* | GAGCGCGTTGTCATTCAGT | CTGGCTGCCATGAGTGAG |
| *Sohlh2* | CATCGAGCTGTTCCTTCCAT | CTGGTCAGCATGGCATCTT |
| *Star* | GGAAGTCCCTCCAAGACTAAAC | TGGTTGATGATTGTCTTCGG |
| *Stra8* | GTGCAAGCTGAACAACAGGA | AGGGACACAGCATTGGAGTC |
| *Trp53* | ACGCTTCTCCGAAGACTGG | AGGGAGCTCGAGGCTGATA |
| *Trp63* | AGACCTCAGTGACCCCATGT | CTGCTGGTCCATGCTGTTC |
